# Zoledronic acid is an effective radiosensitizer in the treatment of osteosarcoma

**DOI:** 10.18632/oncotarget.12281

**Published:** 2016-09-27

**Authors:** Eun Ho Kim, Mi-Sook Kim, Kyung-Hee Lee, Jae-Soo Koh, Won-Gyun Jung, Chang-Bae Kong

**Affiliations:** ^1^ Division of Heavy Ion Clinical Research, Korea Institute of Radiological and Medical Sciences, Seoul 139-706, South Korea; ^2^ Department of Radiation Oncology, Korea Institute of Radiological and Medical Sciences, Seoul 139-706, South Korea; ^3^ Department of Orthopaedic Surgery, Korea Institute of Radiological and Medical Sciences, Seoul 139-706, South Korea; ^4^ Department of Pathology, Korea Institute of Radiological and Medical Sciences, Seoul 139-706, South Korea

**Keywords:** zoledronic acid, radiosensitivity, osteosarcoma cells, apoptosis, DNA damage

## Abstract

To overcome radioresistance in the treatment of osteosarcoma, a primary malignant tumor of the bone, radiotherapy is generally combined with radiosensitizers. The purpose of this study was to investigate a third-generation bisphosphonate, zoledronic acid (ZOL), as a radiosensitizer for osteosarcoma. We found that exposure of KHOS/NP osteosarcoma cells to 20 μM ZOL decreased the γ-radiation dose needed to kill 90% of cells. This radiosensitizing effect of ZOL was mediated through decreased mitochondrial membrane potential, increased levels of reactive oxygen species, increased DNA damage (as assessed by counting γ-H2AX foci), decreased abundance of proteins involved in DNA repair pathways (ATR, Rad52, and DNA-PKcs), and decreased phosphorylation of PI3K-Akt and MAPK pathway proteins (Raf1, MEK1/2, ERK1/2, and Akt), as compared to γ-irradiation alone. Cells treated with ZOL plus γ-irradiation showed impaired cell migration and invasion and reduced expression of epithelial-mesenchymal transition markers (vimentin, MMP9, and Slug). In Balb/c nude mice, the mean size of orthotopic osteosarcoma tumors 2 weeks post-inoculation was 195 mm^3^ following γ-irradiation (8 Gy), while it was 150 mm^3^ after γ-irradiation plus ZOL treatment (0.1 mg/kg twice weekly for 2 weeks). These results provide a rationale for combining ZOL with radiotherapy to treat osteosarcoma.

## INTRODUCTION

Osteosarcomas (OSs) are primary malignant bone tumors identified by the production of osteoid or immature bone [1]. Early treatment paradigms for OS in the 1960s entailed amputation, but modern approaches are multidisciplinary, and have resulted in enhanced overall survival and preservation of organ/limb function [2]. Current treatment approaches generally consist of intensive neoadjuvant chemotherapy followed by resection [3]. Ionizing radiation (IR) therapy may also be part of OS treatment to prevent damaging the surrounding normal tissue [4–6]. However, recent reports have shown that radiotherapy for the local treatment of OS is limited because of radioresistance [5]. Thus, when patients with OS are treated with radiotherapy, it is usually administered in combination with radiosensitizers [6–8].

Bisphosphonates (BPs) are pyrophosphate analogs commonly prescribed to treat osteoclast-mediated bone diseases, including osteoporosis, Paget's disease, malignancy-associated hypercalcemia, bone metastases, and bone disease related to multiple myeloma [9–11]. BPs may influence the differentiation and recruitment of osteoclast precursors or change the ability of mature osteoclasts to resorb bone by altering the permeability of the osteoclast membrane to small ions [12]. BPs are taken up by osteoclasts and induce the specific inhibition of ATP-dependent enzyme systems and apoptosis by inhibiting the mevalonic acid pathway, thereby disrupting the biochemical process that leads to bone destruction. Third-generation BPs, such as zoledronic acid (ZOL), are 10,000–100,000-fold more potent than the older generation BPs [10, 11]. ZOL, which is the most effective clinically available nitrogen-containing BP, has shown efficacy against bone cancer metastasis, demonstrating that nitrogen-containing BPs can decrease skeletal morbidity in both osteolytic and osteoblastic diseases [9, 13, 14].

Recently, preclinical studies have suggested that the combination of ZOL and IR might be an effective anticancer treatment in patients with breast cancer, prostate cancer, myeloma, lung cancer, renal cell cancer, and other cancers [8, 9]. However, it is unknown how ZOL treatment affects the sensitivity of OS to IR. Therefore, we investigated whether ZOL has a radiosensitizing effect on OS cells and primary human cells, *in vitro*, as wells as OS cells *in vivo*.

## RESULTS

### ZOL treatment radiosensitizes human OS cells and orthotopic tumors

**Figure 1:**
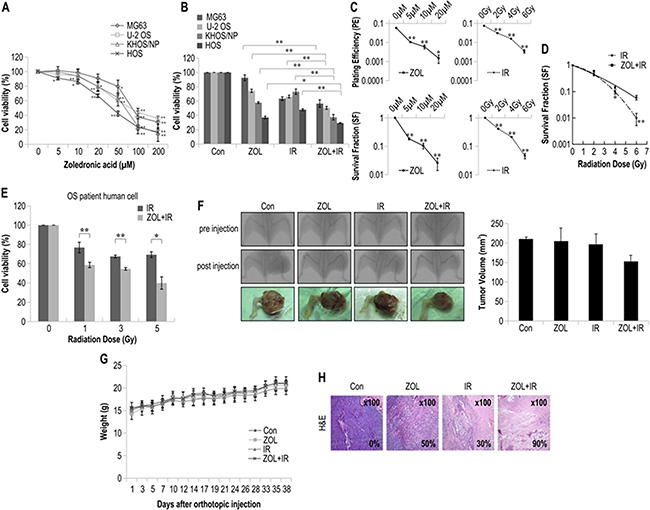
The radiosensitizing effects of zoledronic acid (ZOL) on osteosarcoma (OS) cells treated with irradiation For each assay, values represent the means of 3 experiments ± SD; *p < 0.05, **p < 0.001. **a.** Cell viability of 4 OS cell lines treated with different concentrations of ZOL for 72 h was measured by MTT assay. **b.** Cell viability of 4 OS cell lines treated with ZOL and radiation for 72 h was measured by MTT assay. **c.** The sensitivity of KHOS/NP OS cell lines treated with ZOL and IR was measured by colony-forming assay. The survival fraction, which was expressed as a function of the irradiation dose, was calculated as follows: survival fraction = colonies counted/(cells seeded × PE/100). **d.** The radiosensitivity of KHOS/NP cell lines treated with and without ZOL was measured by colony-forming assay. Data points for γ-irradiated OS cells were fitted with the linear quadratic model. **e.** Cell viability of a primary OS cell line treated with and without ZOL was measured by the MTT assay after irradiation. **f.** KHOS/NP cells were injected into the proximal tibia of 4 groups each containing 3 animals nude mice to generate an orthotopic tumor model. The dimensions of the leg (including the tumor) were measured every 7 days by X-ray analysis. Representative radiographs of the limb of a mouse at 0 and 6 weeks after tumor inoculation are shown. Representative images of animal tumors at 6 weeks and a graph of tumor size against time are shown. **g.** Change in body weight at each time point relative to body weight at the time of treatment. *n* = 3/group, mean ± SD. **h.** Tumors were excised and processed for immunostaining or hematoxylin and eosin staining. Original magnification, ×100.

To examine the effects of ZOL on OS cell lines, we used the MTT assay (Figure 1a). Various doses of ZOL were administered for 72 h to determine the optimal ZOL concentration, and 20 μM ZOL showed the most effective cytotoxicity among the tested concentrations, inhibiting cell growth by about 25% after 72 h exposure. The IC_50_ values for ZOL-treated OS cells are summarized in Table 1.

**Table 1:** IC_50_ values of ZOL-treated OS cells

OS cell line	IC_50_ (μM)
MG63	83
U-2 OS	89
KHOS/NP	67
HOS	43

Using ZOL pretreatment for 24 h, we next investigated the effects of ZOL on IR-induced cell viability in the 4 OS cell lines (Figure 1b). The PE and survival fraction of KHOS/NP cells decreased as the doses of ZOL and IR increased (Figure 1c). The results of the colony-forming assay of γ-irradiated KHOS/NP cells with and without ZOL pretreatment are shown in Figure 1d in the form of survival curves. The experimental survival fraction (S/S0) data points for γ-irradiated OS cells were fitted with the linear quadratic dose (D)-dependent relation given by S/S0 = exp − (αD + βD^2^), where α and β are constants. The fitted values of α and β for γ-irradiated KHOS/NP cells are given in Table 2. Doses of IR required for 90% cell death with and without ZOL were extracted from Figure 1d and are summarized in Table 3. The radiosensitivity enhancement factor values for IR treatment of ZOL-pretreated KHOS/NP cells are shown in Table 4. The values from the fitted curves show that ZOL and IR acted synergistically in reducing the survival fraction, compared with the effect of IR alone (Table 5). The MTT assay showed that ZOL had a radiosensitizing effect on OS primary cells from a patient (Figure 1e).

**Table 2:** Fitting parameters α and β for survival assay data

Cell type	Radiation type	α (Gy^−1^)	β (Gy^−1^)
KHOS/NP	γ-ray	0.348 ± 0.607	0.022± 0.117
	ZOL+γ-ray	0.206 ± 0.569	0.094 ± 0.108

**Table 3:** Radiation dose required for 90% cell death with and without ZOL (data extracted from Figure 1)

Cell type	Radiation type	D10 (−ZOL)	D10 (+ZOL)
KHOS/NP	γ-ray	5.02	3.98

**Table 4:** Radiosensitivity enhancement factor (REF) and dose reduction values

Cell type	Radiation type	REF value	Dose reduction (%)
KHOS/NP	γ-ray	1.26	79

The radiosensitivity enhancement factor (REF) corresponding to 90% cell killing was calculated to quantify the radiosensitization due to ZOL using the equation REF = radiation dose for 90% killing/radiation dose in the presence of ZOL for 90% cell killing.

**Table 5:** Evaluation of the radiosensitization effect of ZOL according to the formula of Valeriote and Carpentier

Radiation Dose	Radiosensitization effect
2 Gy	Synergism
4 Gy	Synergism
6 Gy	Synergism

To investigate the radiosensitizing effect of ZOL *in vivo*, we used an orthotopic model (Figure 1f). ZOL decreased tumor growth in IR-treated mice (Figure 1f). There were no visible signs of toxicity due to ZOL administration in mice, as evidenced by the lack of a difference in body weight (Figure 1g). In addition, H&E staining also revealed that tumors from mice subjected to the combined treatment showed a higher apoptosis rate than those from the mice treated with either IR or ZOL alone (Figure 1h).

### Effect of ZOL on IR-induced apoptosis and cell cycle progression

**Figure 2:**
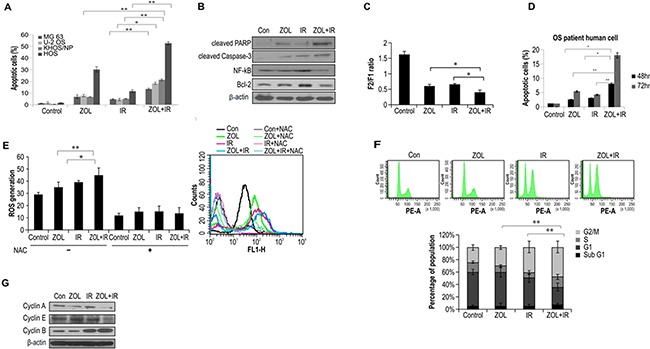
Effects of ZOL and radiation on apoptosis and cell cycle in OS cells Values represent the means of 3 experiments ± SD; *p < 0.05, **p < 0.001. **a.** Four OS cell lines were exposed to a combination of ZOL (20 μmol/L) and 4-Gy radiation. After 48 h, cells were subjected to annexin V and propidium iodide staining and analyzed by flow cytometry. **b.** Cell lysates (30 μg) were immunoblotted with the indicated antibodies. **c.** ZOLchanged the mitochondrial membrane potential of KHOS/NP cells; the mean mitochondrial membrane potentials of each group are provided. **d.** After 48 h, primary OS cell lines were subjected to annexin V and PI staining and analyzed by flow cytometry. **e.** ROS concentration measured at 48 h after treatment with ZOL, IR, and ZOL+IR. **f.** Cell cycle distribution was analyzed quantitatively by flow cytometry. **g.** Cyclin expression was analyzed by western blotting.

To determine the fraction of OS cells induced to undergo apoptosis by the combination treatment, we detected apoptosis by annexin V and PI staining. Notably, 48 h of treatment with ZOL+IR increased the percentage of early apoptotic cells in the 4 OS cell lines (Figure 2a). We also examined whether ZOL enhanced radiotoxicity resulting from caspase-3 activation and PARP fragmentation in KHOS/NP cells (Figure 2b). Both caspase-3 activation and PARP cleavage increased with ZOL+IR treatment compared with treatment with ZOL alone. In addition, we examined the expression of the anti-apoptotic protein Bcl-2 and the cell survival protein NF-κB and found that ZOL+IR treatment decreased the expression of both proteins in KHOS/NP cells (Figure 2b and Supplementary Figure S1a).

The loss of Ψ_m_ is an important step in the apoptotic process and is lethal to cells because it results in the release of various pro-apoptotic factors from the mitochondria into the cytoplasm [15]. The unique cationic dye JC-1 was used to analyze the Ψ_m_ of the mitochondria in KHOS/NP cells after ZOL treatment. There was depletion of Ψ_m_ in KHOS/NP cells treated with ZOL+IR for 24 h when compared with the control (Figure 2c). Exposure to ZOL+IR for 48 h also increased the percentage of early apoptotic cells in primary cells from an OS patient (Figure 2d). These results suggest that ZOL enhances cell death in irradiated cells by decreasing Ψ_m_. Next, we examined the effects of ZOL on ROS production in OS cells. ROS production was more strongly induced by ZOL+IR than by ZOL or IR alone. These data were further confirmed by incubating the cells with *N*-acetylcysteine (NAC), which is a scavenger of ROS. Treatment with NAC almost completely abolished the detection of ROS in KHOS/NP cells (Figure 2e).

To investigate which cellular mechanisms might increase IR-induced cell death following the ZOL treatment, we next examined cell cycle profiles. As shown in Figure 2f, treatment with IR alone resulted in an accumulation of cyclin B1, which is involved in the G2/M transition, whereas ZOL treatment alone increased expression of cyclin E. In contrast, the expression of cyclin A and cyclin E decreased and that of cyclin B1 increased after 24 h of ZOL+IR treatment (Figure 2g and Supplementary Figure S1b).

### Influence of ZOL on IR-induced DNA damage and DNA repair activity

**Figure 3:**
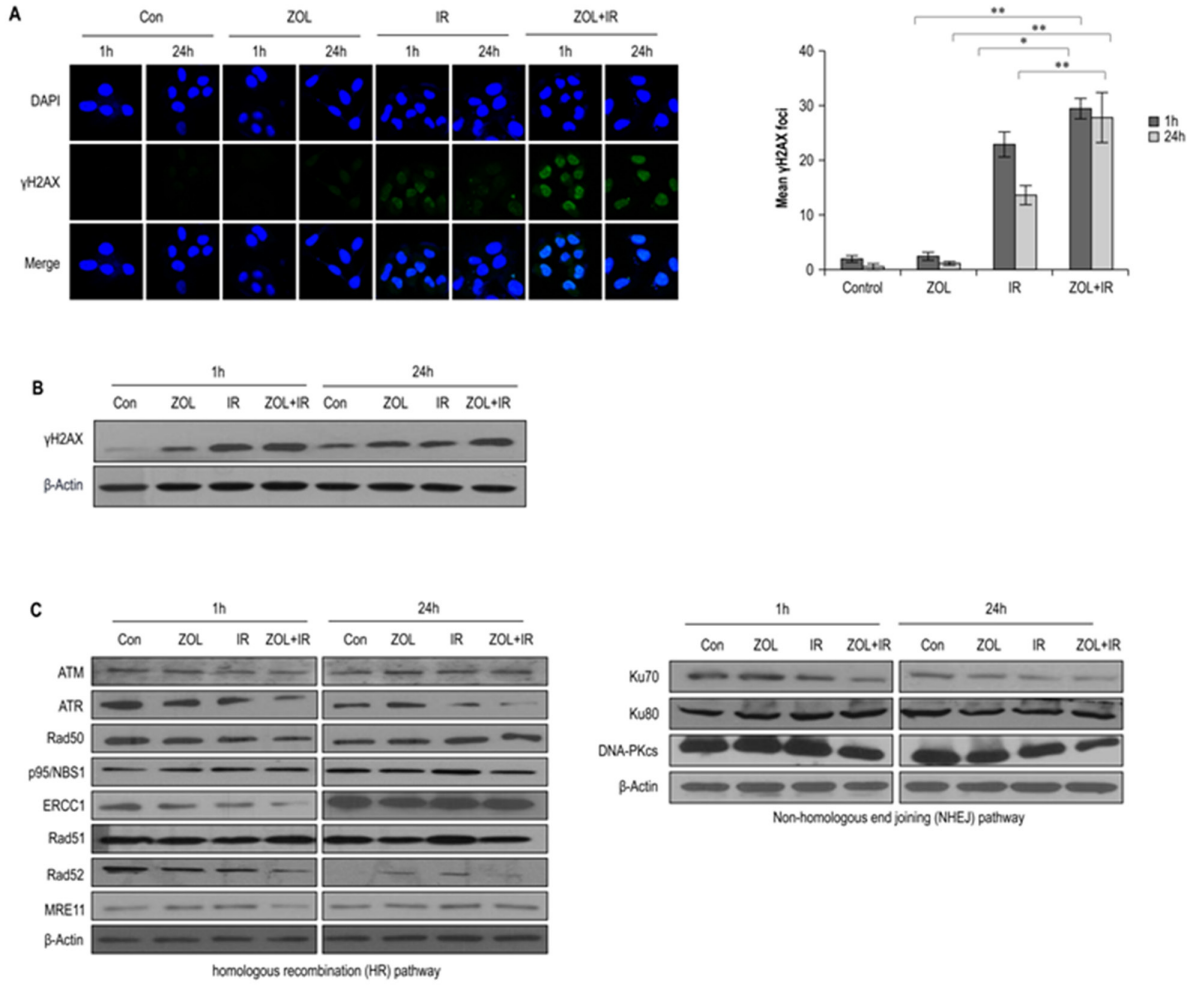
Effects of ZOL on the DNA damage response in irradiated OS cells **a.** Immunocytochemistry for phosphorylated H2AX, a marker of the DNA damage response, in KHOS/NP cells exposed to radiation in the presence of ZOL and analyzed at 1 and 24 h after irradiation. Values represent the means of 3 experiments ± SD; *p < 0.05, **p < 0.001. **b, c.** Immunoblotting of cell lysates with indicated antibodies.

To explore the involvement of cellular DNA repair in ZOL-induced radiosensitization, we evaluated the DNA damage response by analyzing the abundance of the damage-response protein, H2AX, with immunocytochemistry and western blotting. ZOL increases the frequency of IR-induced double-stranded breaks (DSBs), and as expected, ZOL-pretreated cells had higher levels of IR–induced γ-H2AX (Figure 3a, 3b). After ZOL+IR treatment, OS cells showed damaged DNA foci, which appeared 1 h after IR exposure and remained for 24 h (Figure 3a, 3b). In contrast, ZOL treatment alone resulted in the appearance of weak foci from 1 h after the treatment.

To investigate whether the reduced DSB repair activity detected in ZOL+IR-treated cells was due to the suppression of DSB repair-related proteins, we examined the effect of ZOL+IR treatment on the expression of key proteins in the homologous recombination (HR) and non-homologous end-joining (NHEJ) pathways. The relative abundance of DNA damage repair-associated proteins in KHOS/NP cells under various conditions is shown in Figure 3c and Supplementary Figure S2a. The proteins ATR, ERCC1, Rad52, MRE11, Ku70, and DNA-PKcs were downregulated at 1 h by ZOL+IR, and, at 24 h, ATR, Rad52, and DNA-PKcs remained less abundant. This clear downregulation of ATR, Rad52, and DNA-PKcs by ZOL+IR at both time points suggests that DNA damage repair was inactivated by 1 and 24 h exposure to ZOL+IR (Figure 3c and Supplementary Figure S2a). However, only a moderate downregulation of ATM, Rad50, p95/NBS1, ERCC1, Rad51, MRE11, and Ku80 was noted in OS cells treated with ZOL+IR for 24 h. These results suggest that ZOL+IR treatment inhibits the expression of both HR- and NHEJ-related proteins, inhibiting the repair of IR-induced DSBs.

### Effects of ZOL+IR on the abundance of proteins in the PI3K-Akt and MAPK pathways

**Figure 4:**
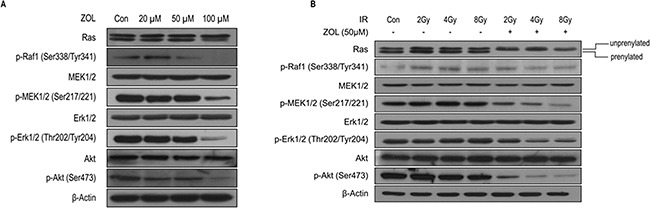
PI3K-Akt and MAPK expression after ZOL and radiation treatment of OS cells **a, b.** Whole-cell lysates from each group were immunoblotted with the indicated antibodies in KHOS/NP cells.

To further clarify the molecular mechanisms by which ZOL radiosensitizes OS cells, we first evaluated the effects of ZOL on the PI3K-Akt and MAPK signaling pathways in KHOS/NP cells (Figure 4a). ZOL inhibits the prenylation of the small G-protein Ras thereby altering the Ras-mediated Erk and PI3K/Akt signaling pathways, which are critical to the intrinsic IR resistance of tumor cells [13, 16, 17]. Concordant with previous reports, ZOL inhibited the prenylation of Ras in a dose-dependent manner (Figure 4a and Supplementary Figure S3a). In KHOS/NP cells, ZOL dose-dependently decreased phosphorylation of MEK1/2, Erk1/2, and Akt (Figure 4a and Supplementary Figure S3a). The next IR treatment resulted in dose-dependent phosphorylation of Raf1, MEK1/2, Erk1/2, and Akt within 24 h (Figure 4b and Supplementary Figure S3b). Phosphorylation of these proteins was noticeably inhibited by ZOL+IR treatment compared with ZOL or IR treatment alone.

### Effect of ZOL and IR on cell motility and cell invasion

**Figure 5:**
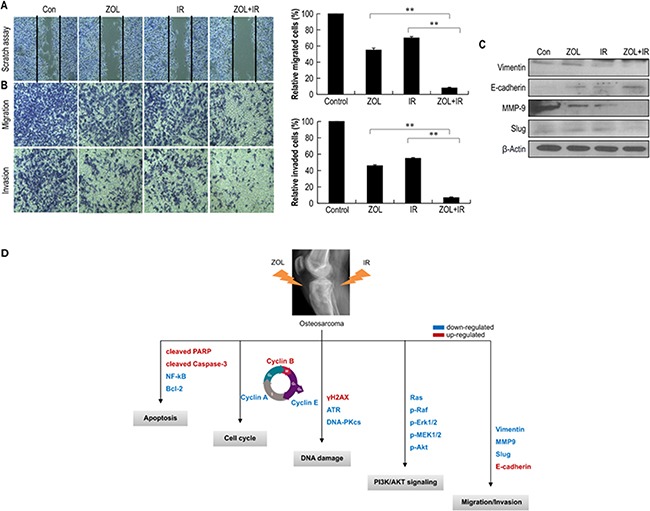
The effect of treatment with ZOL and radiation on the invasion and migration of OS cells Values represent the means of 3 experiments ± SD; *p < 0.05, **p < 0.001 **a.** KHOS/NP cells were scraped with 200-μL pipette tips for the scratch assay and then treated with ZOL and radiation. After incubation for 24 h, the number of cells that migrated across the wound was counted. Each assay was photographed, distances between the migrating cell edges were quantified, and percentage cell migration was calculated. **b.** OS cell invasion and migration after 24-h ZOL+IR treatment were examined by transwell chamber assays. The numbers of invading tumor cells that penetrated through Matrigel^®^ and gelatin were counted in 5 high-magnification microscopy fields. **c.** Cell lysates (30 μg) of OS cells were immunoblotted with the indicated antibodies. **d.** The proposed signaling pathways for the inhibition of invasion, migration, and angiogenesis by ZOL in OS cells.

We next estimated the effects of ZOL+IR on the invasive and migratory abilities of OS cells using a scratch assay. Compared with ZOL or IR alone, treatment with ZOL+IR inhibited cell migration (Figure 5a). We also performed a Matrigel^®^ invasion assay and a gelatin migration assay to examine the effect of ZOL+IR on tumor cell invasiveness, and found that ZOL+IR effectively inhibited tumor cell invasion and migration in OS cell lines (Figure 5b). To investigate the molecular mechanism by which ZOL+IR modulates the expression of epithelial–mesenchymal transition (EMT) markers, EMT proteins and their transcription factors were examined by western blotting. ZOL+IR treatment upregulated the abundance of the epithelial marker protein, E-cadherin, and downregulated the mesenchymal marker protein, vimentin, as well as the transcription factor, Slug, and the effector enzyme, matrix metalloproteinase (MMP) 9, compared with the negative control (Figure 5c and Supplementary Figure S4a). Also, as predicted, ZOL+IR downregulated EMT markers and MMP9, which promote invasion in OS cells (Figure 5c and Supplementary Figure S4a). An overview of the molecular changes induced by ZOL+IR treatment in OS cells is shown in Figure 5e.

## DISCUSSION

Recent reports have elucidated the potential radiosensitizing effects of ZOL in many types of human cancers [8, 9, 18, 19–22]. In the present study, we provide scientific rationale for the clinical application of ZOL as a radiosensitizer in osteosarcoma (OS) by evaluating the effects of single and combined treatments on OS cell survival, apoptosis, cell cycle regulation, DNA repair activity, PI3K-Akt and MAPK signaling, and tumor cell invasiveness/EMT markers *in vitro*, *in vivo*, and in a patient sample. Algur et al. reported that the combined use of ZOL and IR resulted in an enhancement of *in vitro* cytotoxicity beyond that expected for a simple additive effect in two human prostate and myeloma cancer cell lines [22]. However, they did not investigate the underlying molecular mechanisms. Ural et al. previously demonstrated a synergistic cytotoxic effect of ZOL and IR by isobologram analysis, but they also did not investigate the underlying mechanisms [19]. Ryu and Koto revealed that ZOL radiosensitized fibrosarcoma cells and osteosarcoma cells with the induction of apoptosis [20, 21]. However, the detailed mechanisms and *in vivo* effectiveness of ZOL as a radiosensitizer were not elucidated in these studies. Our results suggest that the mechanism underlying the radiosensitizing effect of ZOL appears to be somewhat more complex than what was predicted in these studies.

We demonstrated that ZOL+IR had growth inhibitory effects against human OS cells in an orthotopic model and in primary cells derived from an OS patient by enhancing the radiotoxicity of IR. Orthotopic models are essential for the preclinical evaluation of therapeutic agents, and they are used for studying the pathobiology of tumor progression and metastasis and identifying anticancer agents. In addition, for a model to be accurate enough for predicting the clinical results of a drug treatment, it should replicate the characteristics of the cancer in human patients, including the histological type [23]. Accordingly, we selected an orthotopic model and a culture of OS patient-derived cells. In these models, ZOL showed radiosensitizing effects in combination with IR treatment, as evidenced by increased apoptosis, which manifested as a decreased Ψ_m_, and an increased concentration of ROS.

In addition, when ZOL was administered before IR treatment, OS cells failed to undergo mitosis. This ZOL-mediated inhibition of cell cycle progression appeared to be caused by a failure of the cells to undergo transition from the G2 phase to the M phase, and combined treatment also induced a sub-G1 population. Furthermore, IR treatment results in DNA damage such as DSBs, thereby initiating diverse signaling events in cancer cells [24]. The rationale for using cytotoxic chemotherapy as a radiosensitizer is based on the hypothesis that additional DNA damage would lower the threshold of cell death induced by IR. Thus, to investigate whether ZOL altered DSB repair, we monitored the formation of γ-H2AX foci, a marker for DSBs, in cells treated with ZOL or IR. Our results indicated that an increased number of γ-H2AX foci and high levels of γ-H2AX protein occurred 24 h after IR exposure in the presence of ZOL. Furthermore, ZOL+IR treatment delayed the clearance of γ-H2AX, suggesting that ZOL maintains DNA damage and thus increases the radiosensitivity of cells.

Next, to identify how ZOL suppresses the repair of IR-induced DSBs, we studied the effects of ZOL involved in the NHEJ and HR pathways. Our data showed that ZOL+IR treatment decreased the abundance of ATR kinase and Rad52, which are involved in HR of DNA DSBs, indicating the inhibition of the DNA DSB repair pathway. The abundance of ATM, Rad50, P95/NSB1, ERCC1, Rad51, Rad52, and MRE11 were not altered after 24-h exposure to combination treatment, implying that ZOL induced radiosensitivity by specifically decreasing the abundance of ATR. The abundance of DNA-PKcs protein, which is involved in NHEJ of DNA DSBs, was lower following ZOL+IR treatment than it was after ZOL or IR treatment alone at 24-h exposure, suggesting that ZOL may also sensitize OS cells to IR by interfering with the NHEJ pathway, thus limiting DSB repair in OS cells. Collectively, these results indicate that ZOL might be a dual inhibitor of DNA-PKcs and ATR kinase, thus sensitizing OS cells to IR by inducing DNA damage and inhibiting the repair of IR-induced DNA DSBs.

Many radiosensitizers inhibit PI3K-Akt and MAPK signaling, and this aberrant signaling can promote cell immortalization, proliferation, and radioresistance [13, 16, 17]. Thus, we investigated whether ZOL regulates these signaling pathways to increase radiosensitivity. The dose-dependent phosphorylation of Raf1, MEK1/2, Erk1/2, and Akt within 24 h of IR was suppressed by ZOL. Furthermore, consistent with previous studies, we observed that IR+ZOL-treated OS cells showed a dose-dependent decrease in the abundance of PI3K-Akt and MAPK signaling proteins. Taken together, these results indicate that ZOL increases radiosensitivity by downregulating PI3K-Akt and MAPK signaling.

Finally, we studied the metastatic effects of ZOL+IR treatment. To determine the proteins involved in the inhibition of invasion mediated by ZOL and IR, we evaluated the expression of proteins involved in EMT via western blot analysis. Metastasis occurs through multiple steps, including local invasion, intravasation, transport, extravasation, and colonization [25]. The EMT process has been shown to play a critical role in promoting metastasis in carcinoma [26, 27]. Vimentin is widely used as a marker of EMT, which occurs during metastasis. The expression of MMP9 is also known to increase the invasion and metastasis of tumor cells. The potential roles of MMP9 include the regulation of cancer progression, activation of angiogenesis, and recruitment of macrophages or other bone marrow–derived myeloid cells to the pre-existing metastatic niche [28]. ZOL+IR treatment inhibited the expression of proteins involved in this process, such as vimentin, and suppressed the expression of MMP9, which promotes invasion. Furthermore, ZOL treatment alone suppressed the invasive and migratory capacities of OS cells, and its effect was synergistically increased by treatment with ZOL+IR.

In conclusion, the combination of ZOL with IR increased the efficiency of radiation therapy in OS cells by downregulating signaling pathways involved in DNA DSB repair, cell proliferation/survival, PI3K-Akt/MAPK signaling and cell invasion or migration. For clinical relevance, we used an orthotopic model to further study the radiosensitizing effect of ZOL, and found that ZOL+IR inhibited tumor growth to a greater extent than IR alone did. However, our studies were limited to low-LET IR (γ-radiation). Thus, in future studies we intend to investigate whether ZOL acts as a radiosensitizer for high-LET IR in OS cells. Compared with conventional IR therapy, carbon ion IR therapy based on high-LET IR is effective in treating deep-seated malignant OS tumors. Therefore, further investigation is needed to determine whether ZOL has a synergistic effect with high-LET IR in the induction of OS cell death. Furthermore, it will be necessary to compare the sensitizing effects of ZOL between low-LET (γ-radiation) and high-LET (carbon ion beams) IR to enhance the efficiency and safety of these forms of IR in OS patients. In addition, studies on the effects of ZOL+IR on non-tumor cells should be carried out to minimize the possible complications of their clinical application for the treatment of OS.

## MATERIALS AND METHODS

### Antibodies and chemicals

Anti-NF-κB, anti-Bcl-2, anti-cyclin A, anti-cyclin B, anti-cyclin E, anti-phospho-Raf-1, anti-Akt, anti-Snail, and anti-β-actin were purchased from Santa Cruz Biotechnology (Dallas, TX, USA). Anti-cleaved PARP, caspase-3, anti-ATM, anti-ATR, anti-Rad50, anti-p95/NBS1, anti-Rad52, anti-MRE11, anti-Ku70, anti-Ku80, anti-DNA-PKcs, anti-ERCC1, anti-Rad51, anti-Ras, anti-MEK1/2, anti-phospho-MEK1/2, anti-Erk1/2, anti-phospho-Erk1/2, anti-phospho-Akt, anti-vimentin, anti-E-cadherin, and anti-MMP9 were purchased from Cell Signaling Technology (Danvers, MA, USA), and anti-γ-H2AX was obtained from Millipore (Billerica, MA, USA). ZOL was purchased from Sigma-Aldrich (St. Louis, MO, USA). For *in vitro* experiments, ZOL was dissolved in PBS to make a 2 mmol/L stock solution and stored at −20°C.

### Cell culture and tissue samples

Four OS cell lines were selected for these studies. MG63, U2O2, and KHOS/NP cells were obtained from American Type Culture Collection (ATCC; Rockville, MD, USA) and HOS cells were obtained from the Korean Cell Line Bank (Seoul, South Korea). OS cell lines were maintained in α minimum essential medium (α-MEM; Gibco^®^ Life Technologies, Carlsbad, CA, USA) containing 10% (v/v) fetal bovine serum (FBS; Gibco^®^, Life Technologies) and 1% (v/v) penicillin-streptomycin (Gibco^®^, Life Technologies). OS tissue was obtained with informed consent from a patient who underwent surgery at the Korea Institute of Radiological and Medical Sciences (Institutional Review Board No. K-1603-001-001), and a primary cell culture was established from this tissue. Briefly, tissue was minced into slurry with blades, washed with PBS, then centrifuged for 3 min at 1000 rpm. The supernatant was then discarded and the pellet resuspended in serum-free Dulbecco's modified Eagle's medium (DMEM; WelGene, Daegu, Korea) containing 0.05~0.1% (w/v) collagenase type I (Gibco^®^, Life Technologies) to disaggregate the cells. After 2 h, the cells were washed thoroughly with PBS and maintained in DMEM with 20% (v/v) FBS.

### Irradiation

Cells were plated in dishes and incubated at 37°C and 5% CO_2_ under humidified conditions to 70–80% confluence. Cells were irradiated with a ^137^Cs γ-ray source (Atomic Energy of Canada, Ontario, Canada) at a dose rate of 3.81 Gy/min.

### Colony-forming assay

Cells were pre-incubated with ZOL (20 μmol/L) for 24 h before IR exposure and then incubated for 72 h. After 14–20 days, colonies were stained with 0.4% (w/v) crystal violet (Sigma-Aldrich). The plating efficiency (PE) was the percentage of seeded cells that grew into colonies under the specific culture conditions of the given cell line. The survival fraction, expressed as a function of the IR dose, was calculated as follows: survival fraction = colonies counted/(cells seeded × PE/100). To evaluate the radiosensitizing effects of ZOL, the ratio of the dose (Gy) for IR alone divided by the dose of IR plus ZOL at a survival fraction of 10% was determined. The radiosensitizing effect of ZOL was evaluated according to the formula of Valeriote and Carpentier, as follows:

$$\begin{array}{l} \text{Synergism}:{\text{SF}}_{\text{IR}+\text{ZOL}}<{\text{SF}}_{\text{IR}}X\;{\text{SF}}_{\text{ZOL}}\\ \text{Additivity}:{\text{SF}}_{\text{IR}+\text{ZOL}}={\text{SF}}_{\text{IR}}X\;{\text{SF}}_{\text{ZOL}}\\ \text{Where SF}=\text{survival fraction}. \end{array}$$

### Orthotopic model and histological analysis

Twelve, 4-week old female Balb/c nude mice (average weight 12.1g, range 11.3-13.1g) were obtained from ORIENT Bio (Seoul, Korea) and quarantined for 1 week prior to experimentation. KHOS/NP orthotopic tumors were established as previously described [29]. Briefly, mice were anesthetized by intraperitoneal injection of a mixture of Zoletil (Virbac, Carros, France) and Roumpun (Bayer Korea, Seoul, Korea). The left tibia was wiped with 70% (v/v) ethanol and an 18-gauge needle was inserted through the tibial plateau with the knee flexed, then 1×10^5^ KHOS/NP cells were resuspended in 10 μL PBS and injected into the marrow space of the proximal tibia using a 26-gauge needle coupled to a Hamilton syringe. Two weeks after tumor cell inoculation, mice were randomly assigned into 4 groups of 3 animals each: control group (untreated), ZOL alone group, IR alone group, and combined ZOL and IR group (ZOL+IR). ZOL was administered intraperitoneally twice weekly at a dose of 0.1 mg/kg in 100 μL PBS 2 weeks after inoculation and before and after irradiation, and IR was given as a single dose of 8 Gy (γ-ray using a Co-60 source, 1.99 min/Gy) 2 weeks after inoculation. Animals were euthanized 6 weeks after tumor cell inoculation by CO_2_ asphyxiation. Tumor volumes were determined according to the formula (*L* × *l*^2^)/2 by measuring tumor length (*L*) and width (*l*) with a caliper after euthanasia. All experimental protocols were approved by the Institutional Animal Care and Use Committee of the Korea Institute of Radiological and Medical Sciences. Histological analysis was performed using hematoxylin and eosin (H&E)-stained paraffin sections. [30, 31].

### Detection of apoptotic cells by annexin V staining

After 24 h ZOL pre-incubation, cells were irradiated and subsequently incubated for 48 h. Cells were then washed with ice-cold PBS, trypsinized, and resuspended in 1X binding buffer [10 mm HEPES/NaOH (pH 7.4), 140 mm NaCl, and 2.5 mm CaCl_2_] at 1×10^6^ cells/mL. Aliquots (100 μL) of the cell solution were mixed with 5 μL annexin V/fluorescein isothiocyanate (FITC) (BD Biosciences, Franklin Lakes, NJ, USA) and 10 μL propidium iodide (PI) stock solution (50 μg/mL in PBS) by gentle vortexing, followed by a 15-min incubation at room temperature in the dark. Next, 400 μL of 1X binding buffer was added to each sample, and the samples were analyzed on a FACScan™ flow cytometer (BD Biosciences). A minimum of 10,000 cells was counted for each sample, and data were analyzed using CellQuest™ software (BD Biosciences).

### Western blotting

After ZOL exposure for 24 h, cells were irradiated and cultured for 24 h. Protein from treated cells was extracted with radioimmunoprecipitation assay (RIPA) buffer, separated by SDS-PAGE, and transferred to nitrocellulose membranes. Membranes were blocked with 1% (w/v) nonfat dry milk in TBS with 0.05% (v/v) Tween^®^ 20 and incubated with the indicated antibodies. Blots were incubated with primary antibodies at 1:1000 dilution (5% bovine serum albumin) and secondary antibodies at 1:5000 dilution (5% skim milk). Immunoreactive protein bands were visualized by enhanced chemiluminescence (Amersham Biosciences, Little Chalfont, UK) and scanned. The band intensities were measured using the ImageJ software program (US National Institutes of Health, Bethesda, MD, USA). Data are presented as relative protein levels normalized to β-actin and expressed as a ratio relative to the protein expression of the control samples, which was defined as 1.0.

### Mitochondrial transmembrane potential assay

After pre-incubation with ZOL for 24 h, cells were irradiated, followed by 3 washes with PBS. Next, 1 μM 5,5',6,6'-tetrachloro-1,1',3,3'-tetraethyl-benzimidazolyl-carbocyanine iodide (JC-1; Molecular Probes, Eugene, OR, USA) was added to the cells for 20 min. After JC-1 was removed, the cells were washed with PBS and resuspended in PBS. The amount of JC-1 retained by 10,000 cells/sample was measured at 488 and 575 nm by flow cytometry and analyzed with CellQuest™ Alias software (BD Biosciences). In non-apoptotic cells, JC-1 enters the negatively charged mitochondria, where it aggregates and emits red fluorescence. However, in cells undergoing apoptosis, where the mitochondrial transmembrane potential (Ψ_m_) has collapsed, JC-1 exists as monomers in the cytosol and emits green fluorescence.

### Intracellular reactive oxygen species detection

Reactive oxygen species (ROS) in monocytes were monitored using the fluorescent ROS indicator, C2′,7′-dichlorodihydrofluorescein diacetate (H_2_DCFDA; 5 μM; Molecular Probes). Cell-associated fluorescence was detected by FACS using a FACSort™ flow cytometer with CellQuest™ software (BD Biosciences).

### Analysis of cell cycle progression

Cells were seeded in 60mm dishes at 60% confluency. After 24 h, cells were trypsinized, harvested, and fixed in 1 ml of 70% cold ethanol in test tubes and incubated at 4°C overnight. After incubation, cells were centrifuged at 2,000 rpm for 3 min, and the cell pellets were resuspended in 500 μl propidium iodine (10 μg/ml) containing 300 μg/ml RNase (Sigma, MO, USA). Cell cycle distribution was calculated from 10,000 cells with CellQuest software using FACScaliber (both from Becton Dickinson, CA, USA).

### Immunocytochemistry

Immunocytochemistry was performed to determine the nuclear distribution of γ-H2AX in individual cells. Cells were grown on chambered slides for 1 day prior to IR or ZOL treatment. After 24 h ZOL exposure, cells were irradiated and treated for 1 h or 24 h. All treatments were performed while cells remained attached to the slides, followed by fixation with 4% (w/v) paraformaldehyde and permeabilization with 0.5% (v/v) Triton™ X-100 in PBS. Detection was performed after the slides were blocked in 10% (v/v) FBS/1% (v/v) bovine serum albumin for 1 h with a 1:1000 dilution of FITC-labeled mouse monoclonal antibody against γ-H2AX (Millipore).

### Wound healing (scratch) assay

Human OS cells were seeded onto 6-well plates (Corning) at a density of 2.5×10^4^ cells/well with 3 mL of medium. After ZOL pre-incubation for 24 h, cells were irradiated. On day 2, the monolayers were mechanically disrupted with a sterile 200-μL pipette tip. The assay was performed in duplicate, and wells were photographed every 48 h prior to staining with 0.2% (w/v) crystal violet. Cell migration was monitored using an Eclipse Ti microscope with a DS-Fi1 camera (Nikon, Tokyo, Japan) and migrated cells were counted using ImageJ (US National Institutes of Health).

### Transwell chamber assay

The invasive ability of OS cells was measured using transwell chambers according to the manufacturer's protocol. Briefly, cells were seeded onto the membrane of the upper chamber of the transwell at a concentration of 4×10^5^ cells/mL in 150 μL of medium, and were left untreated or treated with the indicated doses of ZOL, IR, or ZOL+IR for 24 h. The medium in the upper chamber was serum-free, whereas the medium in the lower chamber contained 10% (v/v) FBS as a source of chemo-attractants. Cells that passed through the Matrigel^®^/gelatin-coated membrane were stained with Cell Stain Solution containing crystal violet supplied in the transwell chamber assay (Chemicon, Millipore, Billerica, MA, USA) and photographed after a 24-h incubation period.

### Statistical analysis

Statistical significance was determined by Student's *t*-test. Differences were considered significant if the *p* value was less than 0.05 or 0.001.

## SUPPLEMENTARY FIGURES


