# Supplementary material for: Zoledronic acid is an effective radiosensitizer in the treatment of osteosarcoma
Source: Oncotarget. 2016 Sep 27;7(43):70869–80. doi: 10.18632/oncotarget.12281 (PMC5342595; doi:10.18632/oncotarget.12281)
Supplement: Supplementary file 1 [file oncotarget-07-70869-s001.pdf]

Zoledronic acid is an effective radiosensitizer in the treatment of osteosarcoma

SUPPLEMENTARY FIGURES

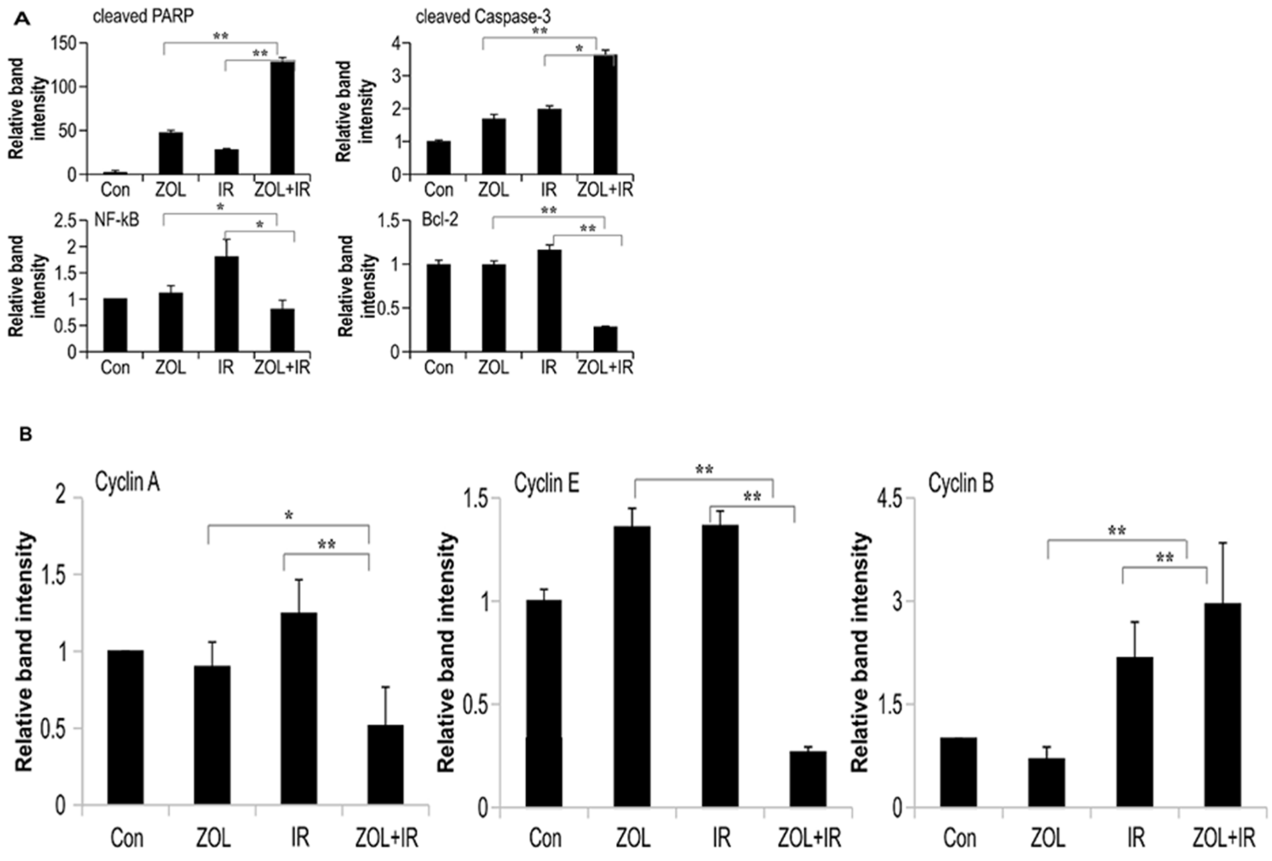

Supplementary Figure S1: Effects of ZOL and radiation on apoptosis and cell cycle in OS cells. a. Band intensities for target proteins were normalized to that for β-actin. Values represent the means of 3 experiments ± SD; *$p < 0.05$, **$p < 0.001$. **b.** Band intensities for target proteins were normalized to that for β-actin. Values represent the means of 3 experiments ± SD; *$p < 0.05$, **$p < 0.001$.

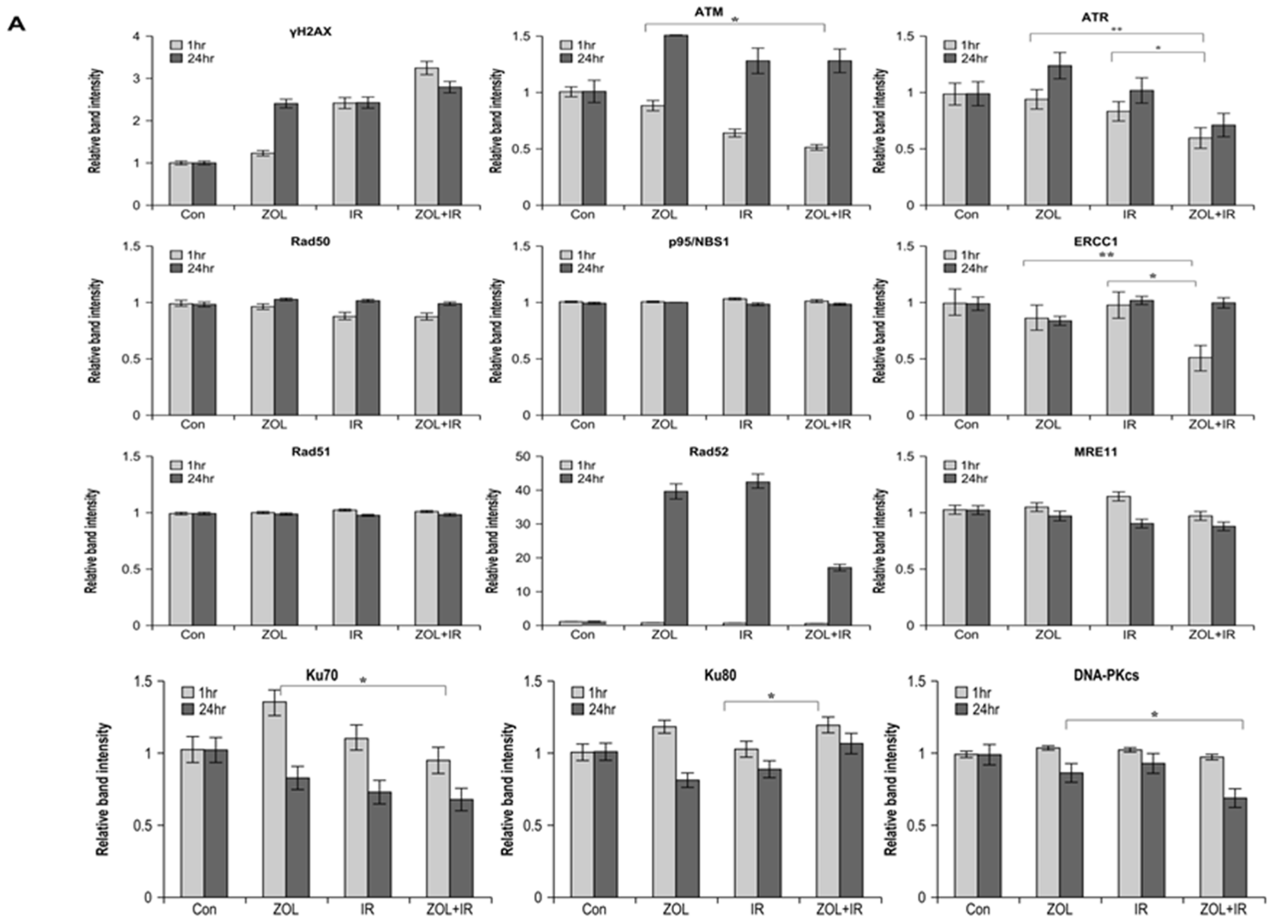

Supplementary Figure S2: Effects of ZOL on the DNA damage response in irradiated OS cells. a. Band intensities for target proteins were normalized to that for β-actin. Values represent the means of 3 experiments ± SD; $*p < 0.05$, $**p < 0.001$.

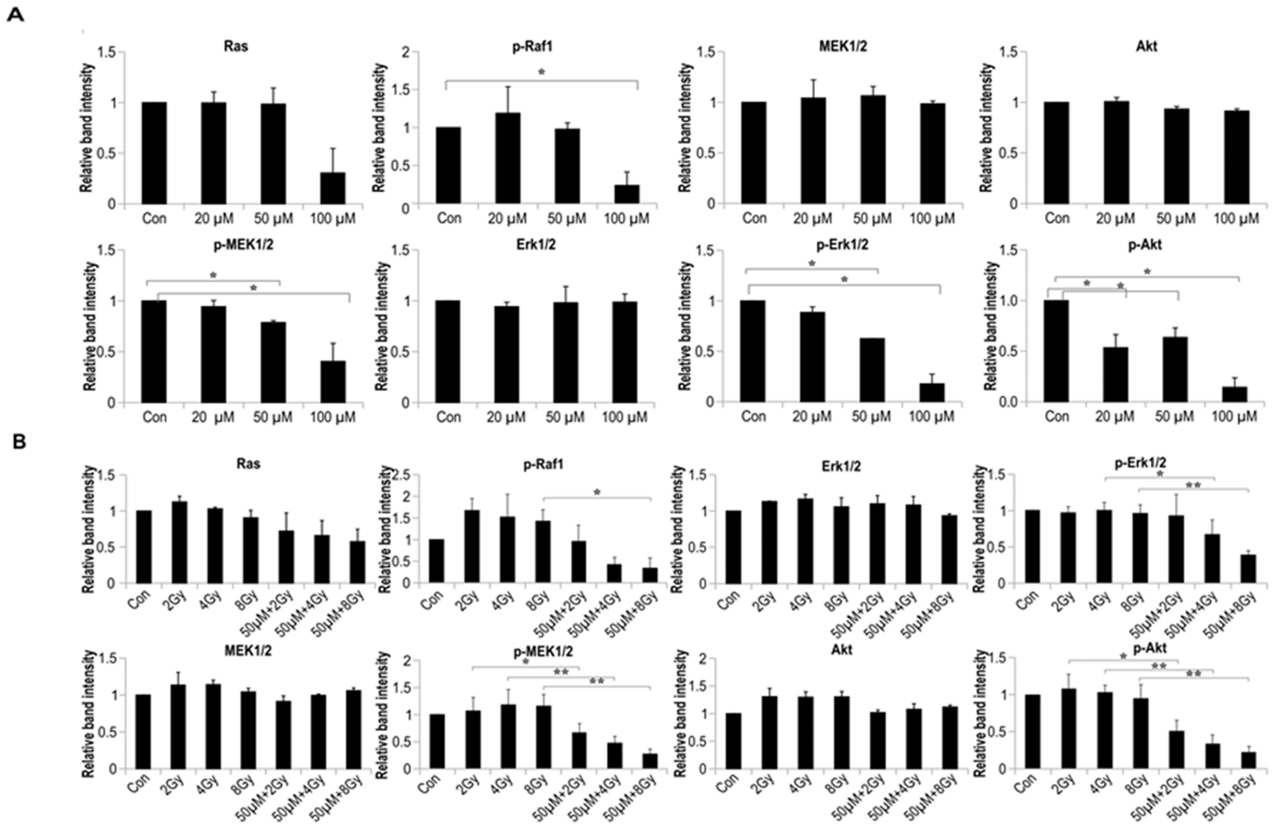

Supplementary Figure S3: PI3K-Akt and MAPK expression after ZOL and radiation treatment of OS cells. a, b. Band intensities for target proteins were normalized to that for β-actin. Values represent the means of 3 experiments ± SD; *$p < 0.05$, **$p < 0.001$.

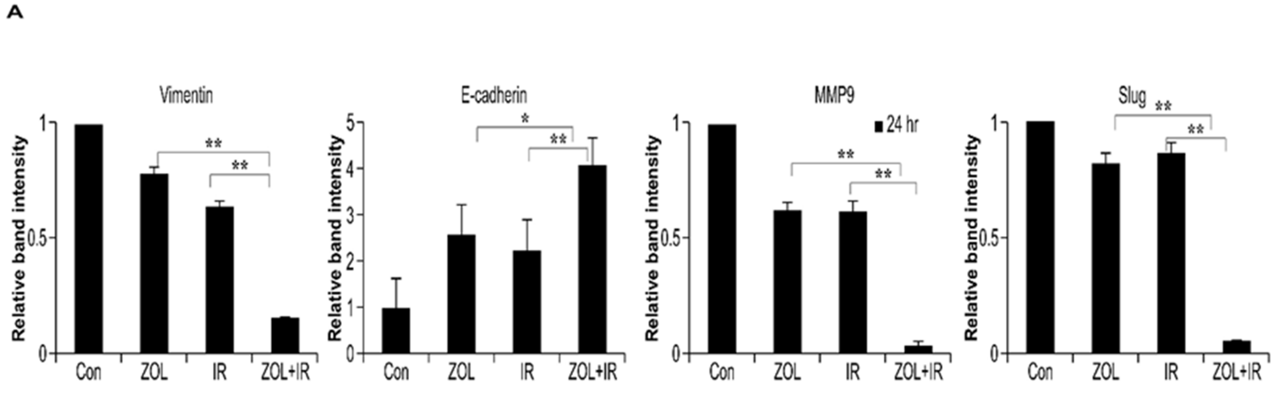

Supplementary Figure S4: The effect of treatment with ZOL and radiation on the invasion and migration of OS cells. a. Band intensities for target proteins were normalized to that for β-actin. Values represent the means of 3 experiments ± SD; *$p < 0.05$, **$p < 0.001$.
